# Recombinant tandem epitope vaccination provides cross protection against *Actinobacillus pleuropneumoniae* challenge in mice

**DOI:** 10.1186/s13568-020-01051-1

**Published:** 2020-07-08

**Authors:** Jiameng Xiao, Jianfang Liu, Chuntong Bao, Rining Zhu, Jingmin Gu, Changjiang Sun, Xin Feng, Chongtao Du, Wenyu Han, Yang Li, Liancheng Lei

**Affiliations:** College of Veterinary Medicine/ Key Laboratory of Zoonosis, Institute of Zoonosis, Jilin University, Xi’an Road 5333, Changchun, 130062 Jilin People’s Republic of China

**Keywords:** Antibody, *Actinobacillus pleuropneumoniae*, Cross protection, Epitope-based vaccine, Prokaryotic expression

## Abstract

*Actinobacillus pleuropneumoniae* (*A. pleuropneumoniae/APP*) is the pathogen that causes porcine contagious pleuropneumonia. *Actinobacillus pleuropneumoniae* is divided into 18 serovars, and the cross protection efficacy of epitopes is debatable, which has resulted in the slow development of a vaccine. Consequently, epitope-based vaccines conferring *Actinobacillus pleuropneumoniae* cross protection have rarely been reported. In this study, B cell epitopes in the head domain of trimeric autotransporter adhesin were predicted, and 6 epitopes were selected. Then, the predicted epitopes (Ba1, Bb5, C1, PH1 and PH2) were connected by linkers to construct a recombinant tandem antigen (*rta*) gene. The RTA protein encoded by the recombinant *rta* gene was expressed, and finally the ICR mice were immunized with the RTA protein with or without inactivated *Actinobacillus pleuropneumoniae* (serovars 1 and 5b) and challenged with *Actinobacillus pleuropneumoniae* to evaluate the protective effect of the epitope-based vaccine and combined vaccine. The mice in the RTA-immunized group and RTA plus inactivated *Actinobacillus pleuropneumoniae* vaccine group had a significant improvement in clinical symptoms and a higher level of antibody in the serum than those in the control group. The RTA immune group had a 40% survival rate after *Actinobacillus pleuropneumoniae* infection, whereas the combination of RTA and inactivated *Actinobacillus pleuropneumoniae* produced very strong cross immune protection in mice, at least 50% (RTA IB1 + C5) and at most 100% (RTA IB5 + C1), whereas no cross immunoprotection was found in the solo *Actinobacillus pleuropneumoniae* immune group. Overall, the combination of the RTA protein and inactivated bacteria significantly enhanced the cross protection effects. This implies that RTA protein in combination with a suitable inactivated *Actinobacillus pleuropneumoniae* strain could be a candidate vaccine for porcine contagious pleuropneumonia.

## Introduction

Porcine contagious pleuropneumonia caused by *A. pleuropneumoniae* is a serious respiratory infectious disease in pigs. It often causes acute outbreaks and is characterized by a high mortality rate, which causes very large economic losses in the global pig industry (Jeffrey et al. 2012). There are 18 serovars of *A. pleuropneumoniae* (Bosse et al. 2018; Liu et al. 2018), and the lack of cross immunoprotection among the major serovars has slowed the development of a vaccine. At present, research on an *A. pleuropneumoniae* vaccine has focused on the pilus protein (Sadilkova et al. 2012), lipopolysaccharide (Li et al. 2018), outer membrane protein (Xie et al. 2016), adhesion (Zhou et al. 2013) and *A. pleuropneumoniae* toxin (Apx) exotoxin (Wu et al. 2018), which are involved in colonization, maintenance, lesion induction and host defense escape (Chiers et al. 2010). Apx toxins can be divided into four types, Apx I-IV; Apx IV exists in all serovars of *A. pleuropneumoniae* strains (Schaller et al. 1999) and is necessary for virulence (Liu et al. 2009). However, due to the complicated structure, the large size of the protein and limited effective epitopes, the subunit structure is not the best option for vaccine development.

Despite the existence of first-generation vaccines against *A. pleuropneumoniae*, the inactivated vaccine still has limitations. Due to the large number of *A. pleuropneumoniae* serovars, heterogeneous serovars cannot provide effective immune protection (Bosse et al. 2002). In the process of vaccine production, certain external factors may also reduce the immunogenicity of the vaccine. Different virulence factors are present in different serotypes, for example, APXI, is present in serovars 1, 5, 9, 10, 11, resulting in different immunization efficacies of inactivated whole-bacterium vaccines (Ramjeet et al. 2008; Seah et al. 2002). Therefore, commercially available inactivated vaccines do not provide complete protection against all serovars. Epitope-based vaccines are a new type of vaccine that has been developed in recent years. The characteristics that make these vaccines good candidates for their development are as follows: (i) Good safety and stability are conducive to convenient transportation and storage. (ii) Epitope-based vaccines can be produced in large quantities by genetic engineering. (iii) Specific antibodies produced against a small epitope can bind to the whole antigen with high specificity. (iv) Epitope-based vaccines can effectively stimulate the production of an effective immune response and avoid the side effects associated with targeting the nonepitope parts of the antigen, for example, hydrophobic amino acid sequences (Yin et al. 2016). However, there are few reports on *A. pleuropneumoniae*-focused epitope vaccine research, which has great potential for development and application.

We previously found that trimeric autotransporter adhesin (TAA) plays an important role in bacterial adhesion, aggregation and biofilm formation, and the head domain of TAA (ADH) is the main functional area (Qin et al. 2017; Wang et al. 2015; Xiao et al. 2012). We also found that *Propionibacterium acnes* (*P. acnes*) could prevent *A. pleuropneumoniae* infection (Lei et al. 2008). Further study showed that the PH1 epitope of Apx IV and the PH2 epitope of a high-affinity periplasmic zinc transporter protein had high homology with a single-stranded DNA-binding protein of *P. acnes*, which had a good immunoprotection effect (Lei et al. 2008). In addition, the cross epitopes Ba1, Bb5, and C1 of *P. acnes* and *A. pleuropneumoniae* screened by phage display technology could effectively prevent *A. pleuropneumoniae* infection (Liu et al. 2017). However, it is still unclear whether the combination of these epitopes has a good immune protection effect.

In this study, the epitopes of ADH were predicted by DNASTAR software and BepiPred 1.0 software, and the protective effect of the epitope-based vaccine was detected. These predicted epitopes were connected to the *P. acnes* antigen epitopes Ba1, Bb5, and C1 and the *A. pleuropneumoniae* epitopes PH1 and PH2 by the GSG linker. The recombinant *rta* gene combined with the *P. acnes* and *A. pleuropneumoniae* epitopes was synthesized, and the RTA protein was expressed by a prokaryotic expression system, purified through nickel column-affinity chromatography and identified by western blotting. Mice were immunized with the RTA protein and challenged with *A. pleuropneumoniae*. Then, the clinical symptoms, weight changes, lung index, number of T cells in the lung, levels of cytokines and survival rates were detected to evaluate the immune protective effect of the RTA protein.

## Methods

### Strains and growth conditions

*Escherichia coli* (E. coli) DH5α competent cells and BL21 (DE3) competent cells (Takara, Japan) were used for cloning and expressing RTA proteins, respectively and were grown at 37 °C in LB medium containing 50 μg/mL kanamycin.

The *APP* serotype 1 reference strain Shope 4074 (*APP 1*, CVCC259) and *APP* serotype 5 reference strain L20 (*APP 5b L20*, CVCC263) were donated by the Shanghai Entry-Exit Inspection and Quarantine Bureau. They were used for the challenge experiment and cultured in brain heart infusion (BHI, USA) medium supplemented with 10 µg/mL NAD (Sigma-Aldrich, USA) at 37 °C for 6 h with shaking at 180 rpm.

### Generation of the target rta gene

TAAs (GenBank: ABN73547.1) were analyzed by daTAA software (https://toolkit.tuebingen.mpg.de/dataa), and the adhesin gene sequences were identified. Then, the DNASTAR and BepiPred 1.0 software programs were used to predict B cell epitopes of the adhesin gene; the epitopes containing α-helixes and β-folds were excluded as much as possible, and the epitopes containing random coils were selected as the dominant B cell epitopes.

These predicted epitopes were connected in series with Ba1, Bb5, C1, PH1 and PH2 by a linker (GSG). Moreover, the restriction enzyme sites EcoR I and Sal I, stop codon TAA and protective bases were added. The target *rta* gene (GenBank: MK603027) was synthesized by BGI-Tech after being converted into *E. coli*-preferred codons.

### Construction of the recombinant vector

Bacteria containing the *rta* gene were cultured overnight to extract the PUC57 plasmid, which was digested by EcoR I and Sal I. Furthermore, the pET28a vector was treated similarly. The *rta* gene and the pET28a vector were recovered via 1% agarose gel electrophoresis and ligated overnight by T4 DNA ligase at 16 °C. The products were transferred into *E. coli* DH5α. After culture expansion, the plasmids were extracted and identified by double enzyme digestion. The identified plasmids were transferred into the *E. coli BL21* (DE3) strain (ice bath 30 min, heat shock 45 s, ice bath 5 min) to express the RTA protein.

### Expression and purification of the RTA protein

Cultures (200 mL) were grown to the exponential phase (OD600 nm = 0.6–0.8) with shaking at 37 °C in LB medium supplemented with 50 μg/mL kanamycin. Bacterial suspensions were induced with a final concentration of 1 mM IPTG and grown at 37 °C. Then, the bacteria were centrifuged at 5000× g for 8 min at 4 °C, washed three times with phosphate-buffered saline (PBS, pH 8.0), and disrupted by sonication (5 s of ultrasound with intervals of 5 s for a total of 30 min) in a mixture of ice and water.

The supernatants were harvested by centrifugation at 10000×g for 10 min at 4 °C and loaded onto a Ni–NTA agarose column (Qiagen, USA). Washing was performed with equilibration buffer, and the RTA protein was eluted with equilibration buffer supplemented with 250 mM imidazole. To remove the imidazole from the samples, the RTA protein was concentrated by ultrafiltration (Millipore, USA) and quantified by a bicinchoninic acid (BCA) protein assay kit (Thermo Fisher Scientific).

### Identification by western blot analysis

The RTA protein was separated by 12% SDS–polyacrylamide gel electrophoresis (SDS-PAGE) and transferred to a PVDF membrane. Subsequently, anti-*APP5b* hyperimmune serum from pigs (stored in our laboratory, 1:5000 dilution) was added to the membrane and incubated for 60 min at 37 °C. Then, the peroxidase (HRP)-conjugated rabbit antibody against swine IgG was diluted (1:5000) and incubated with the membrane. Finally, TMB substrate was added, and the membrane was analyzed with a Tanon 5200 Multi (China).

### Immunization and challenge in mice

For the immunization and challenge experiments, 166 female ICR mice (22–32 g) were purchased from the Animal Experiment Center of Jilin University. The specific laboratory environment was as follows: constant temperature (25 ℃), relative humidity at 40–70%, noise ≤ 60 dB, and artificial lighting (12 h cycle of day and night). All animal feeding and experiments were conducted according to the National Guidelines for Experimental Animal Welfare (Ministry of Science and Technology of China, 2006), and our experimental practices and standards were approved by the Animal Welfare and Research Committee at Jilin University.

Five kinds of vaccines (RTA, RTA + inactivated *APP1*, RTA + inactivated *APP5b*, inactivated *APP1*, and inactivated *APP5b*) were prepared and used in the study. RTA protein, RTA protein and inactivated *APP1* (1 × 10^8^ CFU; *APP1*, inactivated by adding formalin to the medium at a final volume of 0.25%), RTA protein and inactivated *APP5b* (1 × 10^8^ CFU; *APP5b*, inactivated by adding formalin to the medium at a final volume of 0.25%), inactivated *APP1* (1 × 10^8^ CFU) and inactivated *APP5b* (1 × 10^8^ CFU) were mixed with aluminum-gel brine adjuvant (V/V 4:1), respectively. All mice were injected subcutaneously (s.c.) with 0.2 mL of the appropriate vaccine at 0, 14 and 28 d, and nonimmune mice were administered PBS mixed with aluminum-gel brine adjuvant (V/V 4:1) at 0, 14 and 28 d. The details of the groups and treatments are shown in Tables 1 and 2.

**Table 1:** Immunization and challenge treatment groups

Group	Group name	Number of animals	Immunization	Dose	Challenge
1	PBSC1	6	PBS	100 μg/0.2 mL/per mouse	APP1
2	PBSC5	6	PBS	100 μg/0.2 mL/per mouse	APP5b
3	RTAC1	6	RTA	0.2 mL/per mouse	APP1
4	RTAC5	6	RTA	0.2 mL/per mouse	APP5b
5	IB1-C1	6	inactivated APP1	10^8^ CFU of APP1/0.2 mL/per mouse	APP1
6	IB1-C5	6	inactivated APP1	10^8^ CFU of APP1/0.2 mL/per mouse	APP5b
7	IB5-C1	6	inactivated APP5b	10^8^ CFU of APP5b/0.2 mL/per mouse	APP1
8	IB5-C5	6	inactivated APP5b	10^8^ CFU of APP5b/0.2 mL/per mouse	APP5b
9	RTAIB1 + C1	6	RTA +inactivated APP1	100 μg of RTA + 10^8^ CFU of APP1/0.2 mL/per mouse	APP1
10	RTAIB1 + C5	6	RTA +inactivated APP1	100 μg of RTA + 10^8^ CFU of APP1/0.2 mL/per mouse	APP5b
11	RTAIB5 + C1	6	RTA +inactivated APP5b	100 μg of RTA + 10^8^ CFU of APP5b/0.2 mL/per mouse	APP1
12	RTAIB5 + C5	6	RTA +inactivated APP5b	100 μg of RTA + 10^8^ CFU of APP5b/0.2 mL/per mouse	APP5b

Scheme parameters: first detected the level of antibody; recorded the clinical symptoms and the weight changes of mice; 72 h after infection: dissected the mice and weighed the lungs to calculate the lung index; measured the number of T cells of lung by flow cytometry; measure the cytokine level in serum and BALF of the RTA immunization group

**Table 2:** The immunization and challenge methods in the treatment groups in the survival rate experiment

Group	Group name	Number of animals	immunization	Dose of immunization	Challenge
1	PBSC1	10	PBS	0.2 mL/per mouse	APP1
2	PBSC5	10	PBS	0.2 mL/per mouse	APP5b
3	RTAC1	10	RTA	100 μg/0.2 mL/per mouse	APP1
4	IB1-C1	10	inactivated APP1	10^8^ CFU of APP1/0.2 mL/per mouse	APP1
5	IB1-C5	10	inactivated APP1	10^8^ CFU of APP1/0.2 mL/per mouse	APP5b
6	IB5-C1	10	inactivated APP5b	10^8^ CFU of APP5b/0.2 mL/per mouse	APP1
7	IB5-C5	10	inactivated APP5b	10^8^ CFU of APP5b/0.2 mL/per mouse	APP5b
8	RTAIB1 + C1	6	RTA +inactivated APP1	100 μg of RTA + 10^8^ CFU of APP1/0.2 mL/per mouse	APP1
9	RTAIB1 + C5	6	RTA +inactivated APP1	100 μg of RTA + 10^8^ CFU of APP1/0.2 mL/per mouse	APP5b
10	RTAIB5 + C1	6	RTA +inactivated APP5b	100 μg of RTA + 10^8^ CFU of APP5b/0.2 mL/per mouse	APP1
11	RTAIB5 + C5	6	RTA +inactivated APP5b	100 μg of RTA + 10^8^ CFU of APP5b/0.2 mL/per mouse	APP5b

Scheme parameters: Observation of the survival rate

Challenge with *APP* was performed on the 35th day. A single clone of *APP1* or *APP5b* grown on BHI medium was selected and added to 5 mL of BHI liquid medium for growth for 6 h at 37 °C with shaking at 180 rpm. The bacterial solution was centrifuged at 10,000×g for 5 min and resuspended in the original volume with PBS. The bacteria were washed three times with PBS, concentrated and enumerated. Each mouse was anesthetized and then intranasally challenged with 30 μL of *APP1* (6 × 10^8^ CFU) or 30 μL of *APP5b* (4 × 10^8^ CFU) (Table 1). Seventy-two hours after infection, the mice used for sample collection were anesthetized with ketamine hydrochloride (35 mg/kg, intramuscularly (i.m.)) and hydroxylamine hydrochloride (5 mg/kg, intramuscularly (i.m.)), then sacrificed and sampled (Table 1). Mice used for the survival experiment were observed until 72 h after infection, and the survivors were executed by cervical dislocation (Table 2).

### Detection of the antibody level in mouse serum by ELISA

Blood from mice in all groups was separately collected from the tail vein to isolate serum at 0, 14, 28 and 35 d. *APP1* and *APP5b* bacterial suspensions were disrupted by sonication (ultrasound for 5 s at 5 s intervals) until the solution became clear and bright. The protein concentration was determined by the BCA method, and samples were then added to 96-well plates as coating antigens (1 μg per well). The antibody level in the mouse serum in each group was detected by indirect ELISA according to the reference (Yang et al. 2014). When the ratio of the OD value of the positive control serum and the negative control serum was ≥ 2.1, the test results were recorded at 450 nm.

### Observation of clinical symptoms and weight changes in mice

Each mouse was scored according to its clinical symptoms, including appetite, mental state, activity, fur condition, eye secretions, and excreta, at 0, 6, 12, 24, 48 and 72 h after infection. Each clinical symptom of each mouse was scored from 0 to 3. A score of 0 indicated normal, 1 indicated mild symptoms, 2 indicated moderate symptoms, and 3 indicated severe symptoms. The average score of the six clinical symptoms of each mouse was calculated. Finally, the average score of each group was calculated at 0, 6, 12, 24, 48 and 72 h after infection.

The weight of each mouse was measured and recorded at 0, 6, 12, 24, 48 and 72 h after infection. The change in the weight of the mice over time was analyzed.

### Lung index

Mice were sacrificed at 72 h after infection, autopsied, and the lung weight was measured. The lung index of each mouse was calculated according to the formula lung index = lung weight/body weight.

### Detection of the number of T cells in the lungs by flow cytometry

The lungs of the mice were ground into a homogenate, which was filtered through a 200 mesh screen, and then the red blood cells were lysed with red blood cell lysis buffer. Finally, a single-cell suspension was prepared, and according to the instructions of the lymphocyte isolation kit (Tianjin HaoYang Biological Products Company, China), the lymphocytes were separated from the single-cell suspension and enumerated. Then, 1 × 10^6^ lymphocytes were added to a 1.5 mL sterile tube and centrifuged for 10 min at 1250×g. The supernatant was removed, and 500 μL of PBS was added to resuspend the cells. Two microliters of FITC-labeled anti-CD3 antibody (1:250 dilutions) was added under dark conditions, and the mixture was gently mixed and incubated for 30 min at 4 °C under dark conditions. The samples were washed twice with 1 mL of PBS, resuspended in 500 μL of PBS and analyzed as soon as possible by flow cytometry.

### Detection of cytokines

All mice were sacrificed at 72 h after challenge, bronchoalveolar lavage fluid (BALF) and serum were collected, and the levels of cytokines, including IL-1β, IL-6, IL-10, IL-17, IL-18, IL-21, TNF-α, IFN-γ, G-CSF, GM-CSF, Fas-Ligand, and MIP1β/CCL4, in the BALF and serum from each group were detected by indirect ELISA. Detection was performed according to the manufacturer’s instructions (Shanghai, China). The purpose of this experiment was to observe the effect of the RTA protein on cytokine regulation. Samples from groups 1, 2, 3, and 4 were tested.

### Survival rate examination

To confirm the protective effect of RTA protein in infected mice, we examined the survival rate of mice infected with *APP* in which vaccination and challenge were performed as described above and then recorded the survival rates for the analysis of the cross protection ability of RTA protein and inactivated strains at 72 h after infection. The group designations and treatments are detailed in Table 2.

### Statistical analysis

One-way ANOVA and Student’s *t* test were used to compare the differences between different groups. The survival rates of the mice challenged with *A. pleuropneumoniae* were analyzed by the Gehan-Breslow-Wilcoxon test. P-values < 0.05 were considered statistically significant. All statistical analyses were performed using GraphPad Prism 6.0 software.

## Results

### Design of the *rta* gene and prokaryotic expression and purification of the RTA protein

**Fig. 1:**
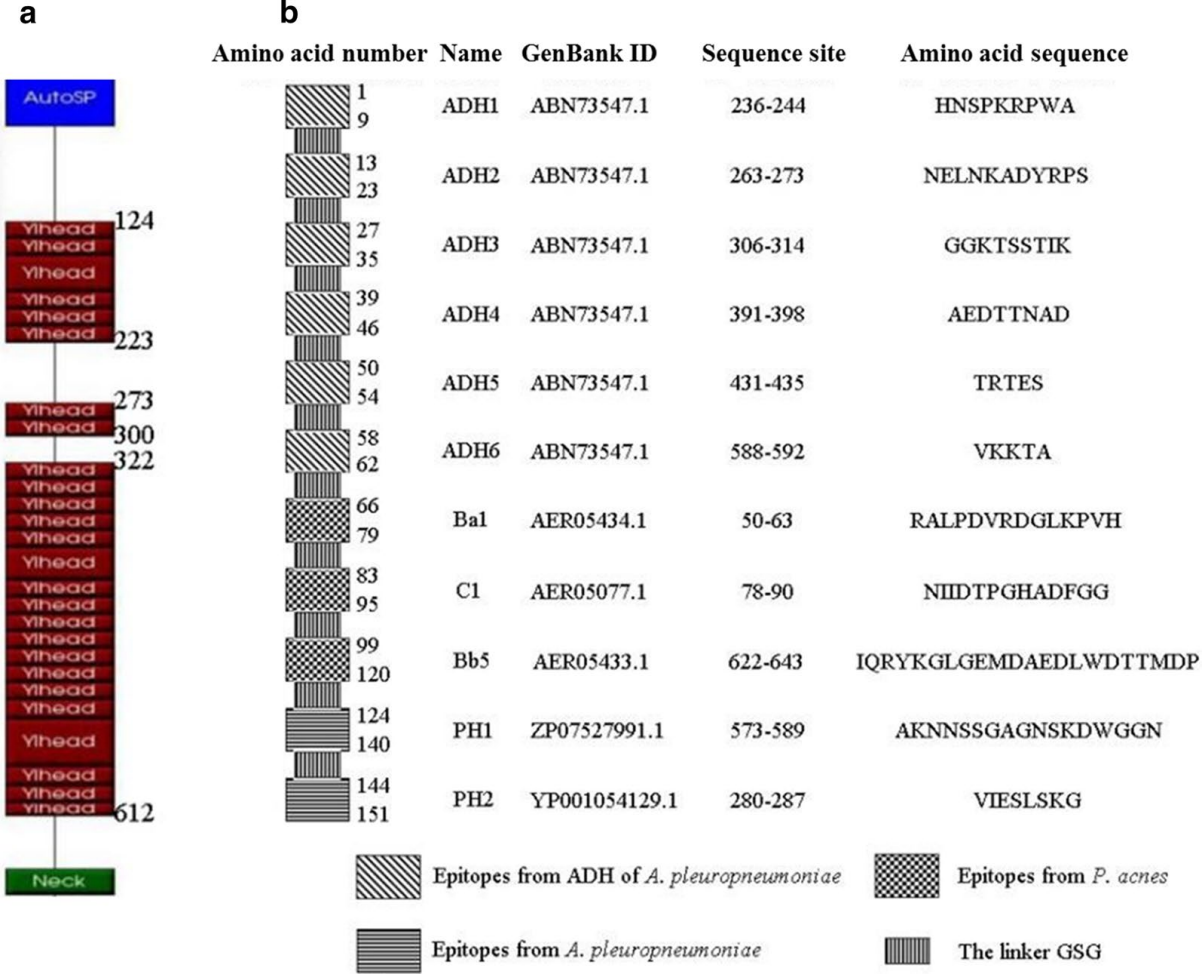
Structural information for the *rta* gene. **a** The composition of ADH was analyzed with daTAA software. **b** Information on the *rta* gene. The epitopes of ADH were predicted by DNASTAR and BepiPred 1.0 software and successively connected with Ba1, C1, Bb5, PH1 and PH2 via a flexible linker GSG

To identify the effective epitope of ADH, TAA was analyzed by daTAA software, which showed that the Ylhead-binding sequence was densely distributed in the 124–612 aa region of ADH (Fig. 1a). Furthermore, the B cell epitope in the 124–612 aa region was predicted by DNASTAR (Additional file 1: Fig. S1A, S1B) and BepiPred 1.0 software. Epitopes containing α-helix and β-fold structures are difficult to produce and were therefore were excluded. Six epitopes with high antigenicity were selected, named ADH1-6, and connected in series with Ba1, C1, Bb5, PH1 and PH2 through a flexible linker (GSG) (Fig. 1b). The optimized nucleic acid sequences were synthesized by BGI-Tech.

**Fig. 2:**
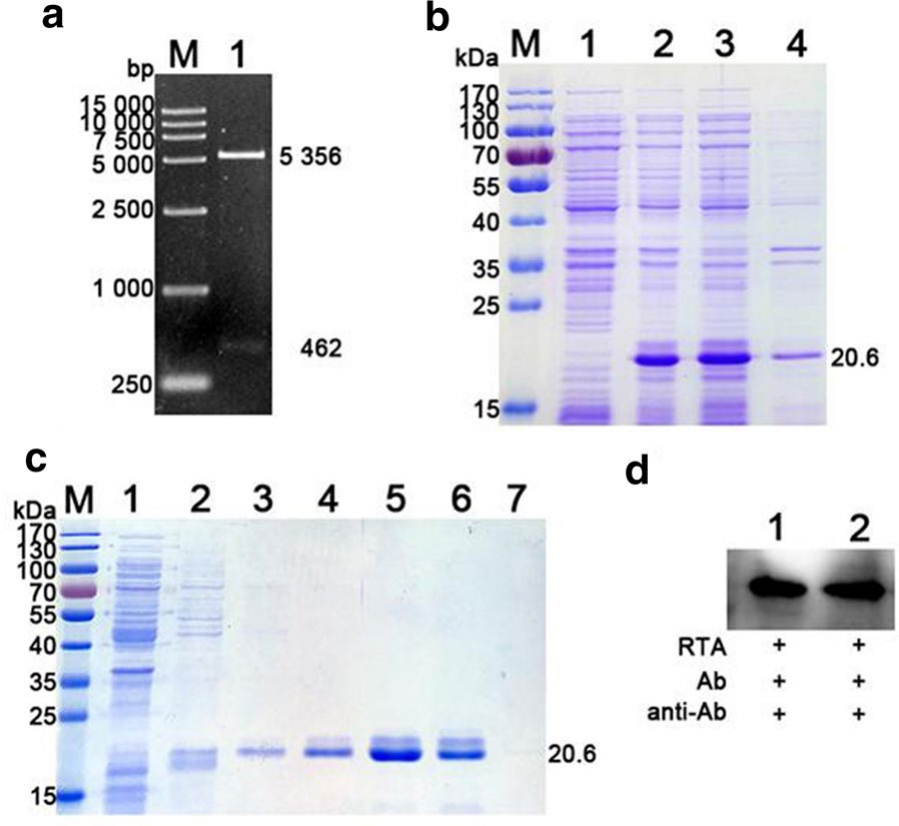
The construction, prokaryotic expression, purification and verification of RTA protein. **a** The electrophoresis result of the pET28a-RTA recombinant plasmid double enzyme digestion. M, 15 000 bp marker; 1, pET28a-RTA recombinant plasmid. **b** The expression of the RTA protein in different fractions of *E. coli* cultures. M, 170 kDa marker; 1, noninduced bacteria; 2, induced bacteria; 3, supernatant after sonication; and 4, precipitate after sonication. **c** The purification of the RTA protein. M, 170 kDa marker; and 1–7, the sample eluted with 10, 20, 40, 60, 80, 100, and 250 mM imidazole, respectively. **d** Western blotting results for the RTA protein. 1, Sample before purification; 2, RTA protein after purification

The *rta* gene and pET28a vector were ligated and transferred into the *E. coli* DH5α strain. The plasmids were extracted and identified by double enzyme digestion. The size of the *rta* gene and pET28a (462 bp and 5356 bp) were predicted, and gel electrophoresis showed that the fragments were within the expected size range (Fig. 2a). The 12% SDS-PAGE results showed that the RTA protein was mainly present in the supernatant rather than in the precipitate (Fig. 2b). To obtain a large quantity of RTA protein, the expression conditions for RTA were optimized. The concentration of RTA protein reached a maximal value after 6–8 h at 37 °C and was unaffected by the inducer (IPTG) concentration (Additional file 2: Fig. S2A–S2C). The RTA protein was expressed under optimal conditions, the cells were lysed by sonication, and the protein was purified by Ni–NTA affinity chromatography. The protein had a predicted weight of 20.6 kDa, and SDS-PAGE showed that the expressed protein was at the expected weight; thus, the relatively pure target protein was obtained (Fig. 2c). The reactivity of the RTA protein was verified by western blotting. The results showed that the RTA protein was specific enough to be recognized by anti-*APP5b* hyperimmune serum (Fig. 2d), indicating that the RTA protein had good reactivity.

### A high level of cross-serotype antibody is produced by combined immunization

**Fig. 3:**
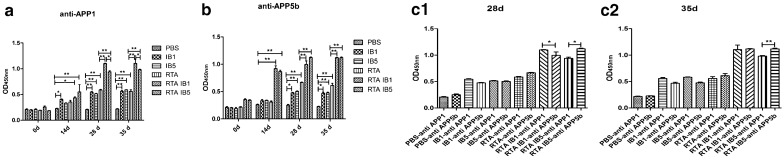
The serum antibody level of the mice in all groups was detected by indirect ELISA. Each group had 6 mice. **a***APP1* was used as the coating antigen. **b***APP5b* was used as the coating antigen. **c1** At day 28 of immunization, the antibody level of the mice in all groups was detected by indirect ELISA. **c2** At day 35 of immunization, the antibody level of the mice in all groups was detected by indirect ELISA

The serum antibody level before challenge was detected by indirect ELISA using *APP1* (Fig. 3a) or *APP5b* (Fig. 3b) as the coating antigens and reading the OD value at 450 nm. The results showed that the level of antibody gradually increased even though the serum from each group was diluted 10 times and whole vaccinated groups showed higher levels of specific antibody than the PBS group (P < 0.05 or P < 0.01), almost reaching a peak at the 28 d post immunization time point. To our surprise, compared with the groups immunized with inactive *APP* alone, all mixed-antigen-immunized groups (RTA + IB1 or IB5) showed dramatic increases in the levels of *APP1*- or *APP5b*-specific antibodies after 28 d (P < 0.01), but there was no difference between the groups immunized with RTA alone and *APP* alone (Fig. 3a, b). In addition, compared with the mice in the PBS group, the mice in the inactivated *APP1* strain-immunized group produced higher levels of specific antibodies against both *APP1* and *APP5b*; similarly, the mice immunized with the inactivated *APP5b* also produced higher levels of antibodies against both *APP5b* and *APP1*, but the cross reactivity of the serum was not equal to that against the same serotype, P < 0.05 at 28 d and P < 0.01 at 35 d (Fig. 3c1, c2). These results show that mice in the RTA immunization group produced many specific cross-reactive antibodies against *APP1* or *APP5b,* and the mixture of RTA with inactivated *APP* produced extremely strong specific antibodies and distinctly moderately reactive cross-reactive antibodies.

### RTA protein vaccination alleviated the clinical symptoms of mice infected with *APP1* or *APP5b*

**Fig. 4:**
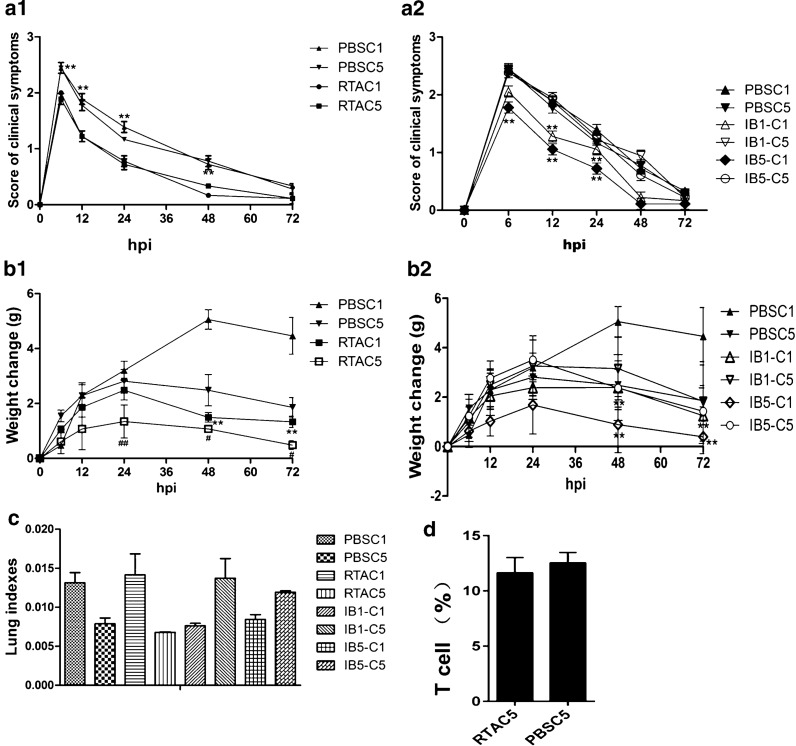
Detection of the clinical symptoms, weight changes, lung indexes, and number of T cells in the lungs of the mice from different groups. Each group had 6 mice. **a1**, **a2** The clinical symptoms of each mouse were observed and scored at 0, 6, 12, 24, 48 and 72 h after challenge. **b1**, **b2** The weight of each mouse was recorded at 0, 6, 12, 24, 48, and 72 h after challenge, and the weight changes of the mice were calculated (weight changes = the initial weight- the real-time weight); * represents the RTAC1 group or the IB5-C1 group or the IB1-C1 group compared with the PBSC1 group and was statistically significant; # represents the RTAC5 group or the IB1- C5 group or the IB5-C5 group compared with the PBSC5 group and was statistically significant. **c** The body weight and the lung weight of the mice were recorded at 72 h after challenge, and the lung indexes of the mice were calculated. **d** The mice in the RTAC5 and PBSC5 groups were challenged with *APP5b*. The number of T cells in the lungs was detected by flow cytometry at 72 h after challenge. * or #, P < 0.05; ** or ##, P < 0.01

The clinical symptoms of the challenged mice in each group were observed. The clinical symptoms of the mice in the RTA immunization group were more significantly alleviated than those of the mice in the PBS control group at 6, 12, 24, and 48 h after infection with *APP1* or *APP5b* (P < 0.01, Fig. 4a1), hinting that RTA protein provides cross protection in vivo. In particular, compared with the PBS control groups, the inactive *APP1* group and inactive *APP5b* group showed a dramatic decrease in clinical symptom scores only when challenged with *APP1* and *APP5b,* respectively (P < 0.01, Fig. 4a2), but no differences were noted in the IB1-C5 and IB5-C1 groups, suggesting that inactivated *APP* cannot induce clear cross protection. Additionally, there was no significant difference between the RTAC1, RTAC5 and IB1-C1, IB5-C5 groups.

The weight of each mouse was measured at 0, 6, 12, 24, 48 and 72 h after challenge, and the weight change in the mice was observed. The results showed that the mice in all of the groups rapidly lost weight within 24 h after the challenge, and then the trend abated. Notably, the RTA immunization groups had significantly greater body weights at 48 h and 72 h (P < 0.01) than the PBS control groups; in particular, the RTAC5 group displayed the least weight change (less than 1 g), which was less than that in the PBSC5 group (approximately 2.5 g) at 72 h (Fig. 4b1). Slight weight changes were observed in the IB1-C1 and IB5-C5 groups compared with the PBS control groups at 48 h and 72 h (P < 0.01, Fig. 4b2).

Moreover, infection with *A. pleuropneumoniae* causes pulmonary edema in animals, which can be evaluated through the detection of the pulmonary indexes in mice. There was no significant difference in the lung indexes between the groups. However, compared with that in the PBSC5 group, the degree of pulmonary edema in the RTAC5 group showed a downward trend (Fig. 4c).

Furthermore, host T lymphocyte populations have been reported previously during *APP* infection; therefore, to evaluate the immune status of the mice at 72 h after challenge, the number of T lymphocytes in the lungs of the mice was detected by flow cytometry. The results showed that there was no significant difference in the number of T lymphocytes in the lungs between the RTAC5 group and the PBSC5 group (Fig. 4d).

These results indicate that the RTA protein plays a protective role by alleviating the symptoms of challenged mice, and the effect does not depend on the T cell-mediated immune response. Moreover, the increased antibody level suggests that the protective effect of the RTA protein likely depends on the B cell-mediated immune response.

### RTA protein vaccination reduced the level of cytokines produced by mice after *APP* infection

Because proinflammatory cytokines usually play an important role in pneumonia caused by microbes, the levels of cytokines in the BALF and serum of each group were detected by indirect ELISA at 72 h post challenge in samples from sacrificed mice.

**Fig. 5:**
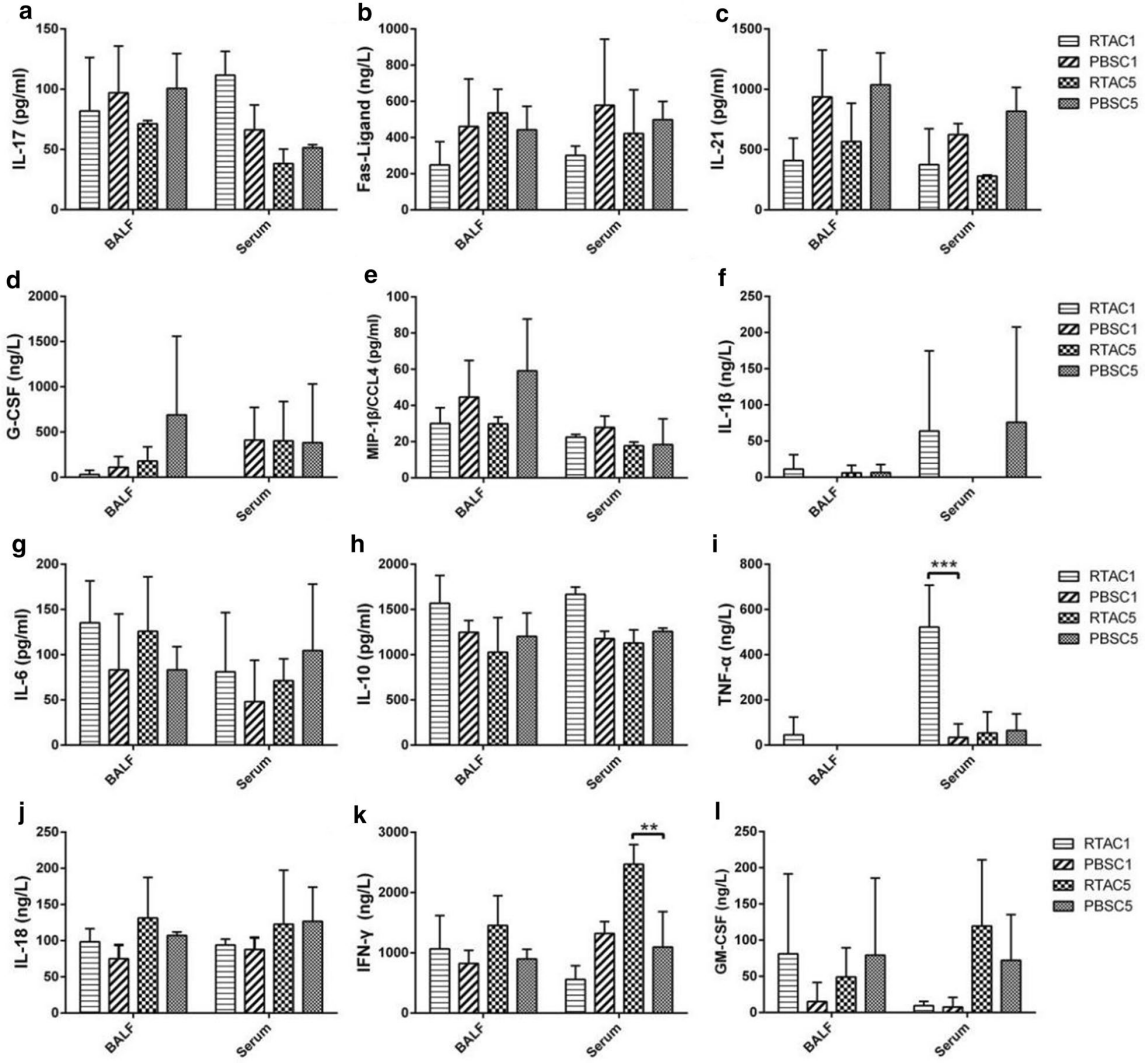
Detection of the cytokine level in the BALF and the serum of mice at 72 h after challenge. The changes in the expression levels of cytokines in the BALF and serum of each group were detected by indirect ELISA, namely, the changes in IL-17 (**a**), Fas-ligand (**b**), IL-21 (**c**), G-CSF (**d**), MIP-1β/CCL4 (**e**), IL-1β (**f**), IL-6 (**g**), IL-10 (**h**), TNF-α (**i**), IL-18 (**j**), IFN-γ (**k**) and GM-CSF (**l**) levels. *, P < 0.05; **/***, P < 0.01

The results showed that although there was no significant difference in the cytokine levels, the average level showed a robust change trend distinguishing the vaccinated and PBS control groups. Based on the average level, the IL-17, IL-21, G-CSF and CCL4 levels in the BALF and the Fas-Ligand, IL-21, G-CSF, and CCL4 levels in the blood of the RTA group were generally lower than those of the PBS group, whereas the IL-6, IL-18, and IFN-γ levels in the BALF and TNF-α in the blood of the RTA group were higher than those of the PBS group. There was no consistent change trend in the levels of other cytokines (Fig. 5a–l). These results indicate that the RTA protein response could decrease the level of inflammatory cytokines and improve the level of anti-inflammatory cytokines at 72 h after *APP* challenge, when the levels of multiple inflammatory cytokines were extensively increased in the lung, thus playing an immunoprotective role in the pathogenesis of *APP*. Furthermore, the levels of certain cytokines, such as TNF-α and IFN-γ, were distinctly changed in lungs and peripheral blood (Fig. 5i, k) These results suggest that cytokines that constitute a complex network in animals that differs in the lungs and blood, and the inflammatory response is the result of the interaction of many anti-inflammatory and proinflammatory cytokines.

### Vaccination with the mixed RTA protein and inactive strain conferred strong cross protection to mice

To evaluate the effect of the RTA protein on the survival rate of mice, *APP1* was used to challenge the mice immunized with the RTA protein, and the survival rates of the mice were recorded. The results showed that compared with that in the PBS control mice, the time to death in the mice treated with the RTA protein was extended within the first 12 h, and the protection rate in the RTA-treated mice was only 40% (Additional file 3: Fig. S3). This implies that the RTA protein has a limited protective effect on mice infected with a lethal dose of *APP1*, but the effect was not sufficient to maintain a high survival rate. RTA immunization can significantly reduce the clinical symptoms of mice infected with serotype 1 and serotype 5, and its effect resembles that of the inactivated strain against homoserotypes (Fig. 4). Therefore, we mixed RTA with inactivated *APP1* and *APP5b* bacterial proteins, immunized mice for 35 d to be challenged with *APP1* or *APP5b*, and recording the clinical symptoms and survival rates (Tables 1 and 2).

**Fig. 6:**
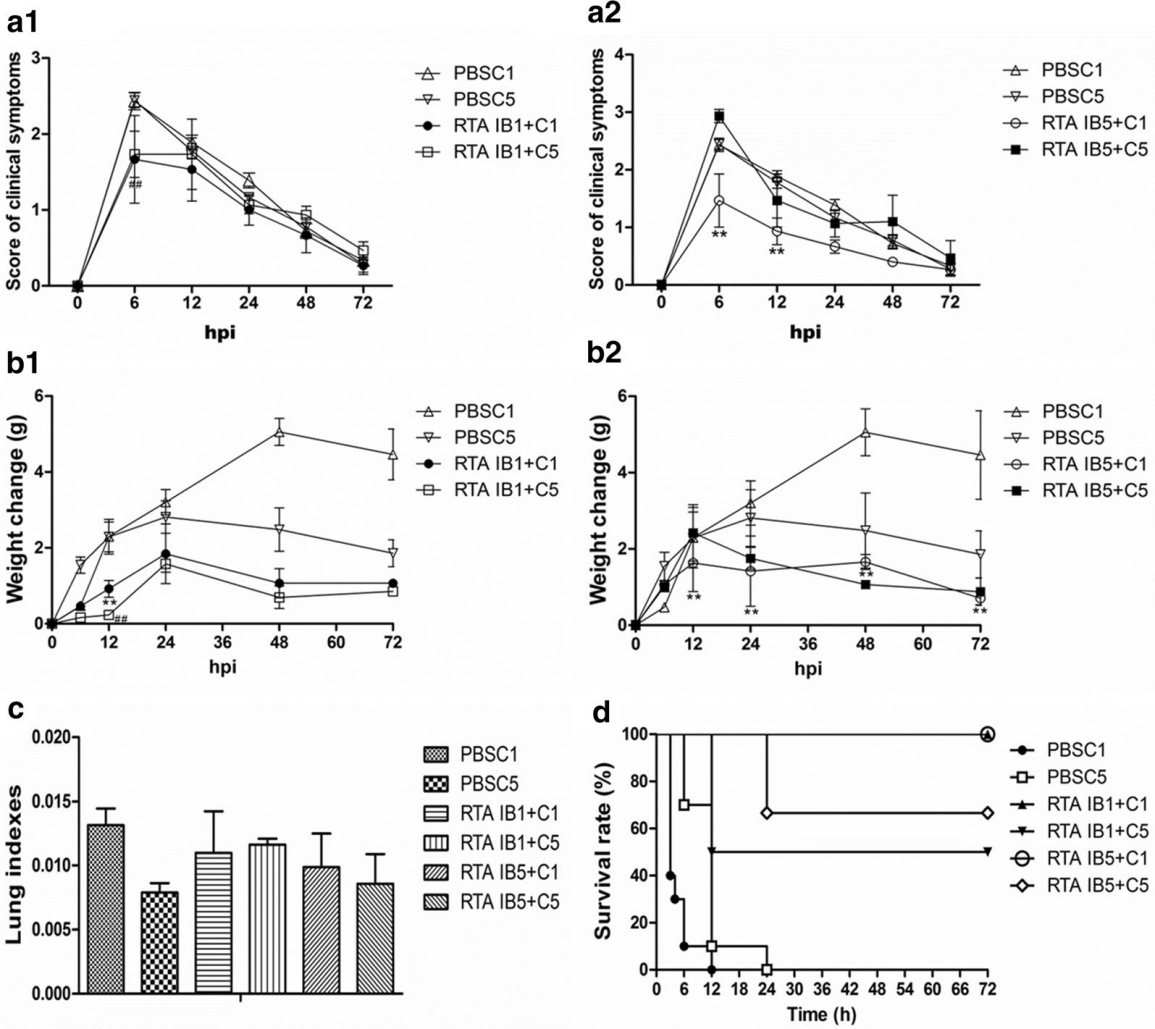
Detection of the clinical symptoms, weight changes, lung indexes and survival rates of mice. **a1**, **a2** The clinical symptoms of each mouse were observed and scored at 0, 6, 12, 24, 48 and 72 h after challenge. **b1**, **b2** The weight of each mouse was recorded at 0, 6, 12, 24, 48, and 72 h after challenge, and the weight changes of the mice were calculated (weight changes = the initial weight- the real-time weight); * represents the RTA IB1 + C1 group or the RTA IB5 + C1 group compared with the PBSC1 group and was statistically significant; # represents the RTA IB1 + C5 group or the RTA IB5 + C5 group compared with the PBSC5 group and was statistically significant. * or #, P < 0.05; ** or ##, P < 0.01. **c** The body weight and the lung weight of the mice were recorded at 72 h after challenge, and the lung indexes of the mice were calculated. **d**. The survival rates of the mice immunized with PBS, RTA protein combined with inactivated *APP1* or *APP5b* at 0, 14, and 28 d and challenged with *APP1* and *APP5b* separately at 35 d. The mice were observed for 3 d to record the survival rate

The clinical symptoms of the combined immunization groups were generally mild. The clinical symptoms of the RTA IB1 + C5 group were significantly less severe than those of the PBSC5 control group (P < 0.01) at 6 h after challenge with *APP5b*, and the clinical symptom scores of the RTA IB5 + C1 group were significantly lower than those of the PBSC1 control group (P < 0.01) at 6 h and 12 h after *APP1* infection (Fig. 6a1, a2). In the combined immunization group, body weight loss was significantly reduced after infection. Compared with those in the PBSC1 or PBSC5 group, mice in the RTA protein plus *APP1* vaccination group lost very little body weight at 6 h regardless of *APP1* or *APP5b* infection (P < 0.01, Fig. 6b1): furthermore, the immunization with RTA plus *APP5b* significantly reduced the body weight loss in mice at 12 h, 24 h, 48 h and 72 h (P < 0.01, Fig. 6b2).

In addition, the degree of pulmonary edema in all coadministration groups showed a downward trend, but there was no significant difference (Fig. 6c).

The survival experiments showed that the protection rate of mice immunized with RTA and inactive *APP1* was 100% after challenge with *APP1* (RTA IB1 + C1 group) and 50% against *APP5b* (RTA IB1 + C5 group); the protection rate of mice immunized with RTA and inactivated *APP5b* was 66% (RTA IB5 + C5 group) against *APP5b* but 100% against *APP1* (RTA IB5 + C1), and no cross immunoprotection was found in the sole *APP* immune group (Fig. 6d, Additional file 3: Fig. S3). This result shows that compared with the individual antigens, the combination of RTA and inactivated *APP* can produce very strong immune protection, at least 50% (RTA IB1 + C5) and at most 100% (RTA IB5 + C1). Overall, the combination of the RTA protein and inactivated bacteria significantly enhances the cross protection effects, especially the combination of RTA and *APP5b,* which was the best antigen for serotype cross protection.

## Discussion

Porcine contagious pleuropneumonia is a serious threat to the modern pig industry, but vaccine development for this disease is slow due to the lack of cross protection induced by inactivated vaccines, and many studies have focused on the subunit structure. However, due to the complex subunit structure, there are few effective antigenic epitopes exposed on the surface of the protein that could cause a cross immune-protection response. Therefore, the epitopes of ADH were predicted by software and connected in series with the Ba1, C1, Bb5, PH1, and PH2 epitopes found by our group through a flexible linker (GSG) to form an epitope antigen. Then, the RTA protein was expressed, purified, and used to immunize mice that were subsequently challenged with *A. pleuropneumoniae*. Our results showed that the RTA protein could alleviate the clinical symptoms, regulate the levels of antibodies and cytokines, such as TNF-α and IFN-γ, and had a limited protective effect against *APP* infection; however, RTA proteins plus inactivated *APP* produced dramatic cross protection. This implies that RTA with a suitable inactivated *APP* strain could be a candidate vaccine for porcine contagious pleuropneumonia.

All the epitopes were short peptides with low molecular weights, which made their immunogenicity extremely low. To enhance the immunogenicity of these epitopes, they were connected head to tail with a GSG linker. The addition of a flexible linker was of great importance for increasing the immunogenicity of the polypeptide chain. Furthermore, the linker could reduce intermolecular interactions and maintain the protein structure. Yano et al. used two lysine residues as linkers to connect T cell epitopes and B cell epitopes to solve the problem of MHC restriction (Yano et al. 2005). RTA was successfully constructed using a GSG linker and induced a better immune response than other antigens, but we should perform further studies on the linker to improve its antigenicity.

In addition, we found that ADH-immunized mice had a 40% survival rate when infected with a lethal dose of *APP5b* (Xiao et al. 2012), whereas immunization with Ba1 and Bb5 produced 80% and 90% survival rates, respectively, when in mice infected with *APP1* and *APP5b* (Liu et al. 2017). *P. acnes* Ssb, which has a high level of homology with PH1 and PH2, produced 80% and 60% survival rates in mice infected with *APP1* and *APP5b*, respectively (Li et al. 2013). In this study, the RTA protein produced a 40% protection rate in *APP*-infected mice. This result may be due to the high proportion of ADH epitopes in the RTA protein, whose protective effect was not as good as that of other polypeptide epitopes. The reason may also be that the selected linker is not optimal and causes changes to the RTA protein spatial structure due to intermolecular forces, thus blocking some of the key epitopes in the middle of the protein. In addition, the epitopes in the RTA protein are linked together, which limits the activity of each epitope and prevents specific immune responses to each epitope, thus reducing the protective effect of the RTA protein. This problem can be effectively overcome by immunizing mice with the combination of the RTA protein and inactivated bacteria, especially coimmunization of RTA with inactivated *APP5b,* which produced a 100% survival rate in mice challenged with *APP1*. This finding implies that choosing a suitable *APP* strain to mix with RTA may be a better way to improve candidate *APP* vaccines in the future.

There are many reports in which the current mouse model was used to evaluate novel *A. pleuropneumoniae* vaccines. For example, Shao et al. evaluated the immunological effect of recombinant Apx toxins and the recombinant outer membrane protein (OMP) (Shao et al. 2010). Park et al. used a murine model and showed that the M cell-targeting ligand-conjugated ApxIIA toxin fragment induces protective immunity against *A. pleuropneumoniae* infection (Park et al. 2015). In addition, we found that the clinical symptoms of mice infected by *APP* are similar to those of infected piglets when we researched the *APP* virulence factor ADH. This result indicates that the results obtained from the mouse model played an important role in guiding *APP* infection research in pigs (Wang et al. 2016). Therefore, a mouse model can provide an important reference and information for researching the pathogenesis mechanisms of *APP* and developing vaccines against *APP*.

At present, most attention is paid to the ApxIV recombinant subunit vaccine, which also has cross protection, but other antigens, such as pili, could be helpful for producing immune protection (Shao et al. 2010). Current commercial vaccines cannot provide complete protection because the fixed antigen that induce complete immune protection have not been identified (Rycroft and Garside 2000). Some scholars have found that the recombinant ApxIV protein combined with inactivated *APP1*, known as a multicomponent vaccine, has a good protective effect against both homologous and heterologous *A. pleuropneumoniae* (Wu et al. 2018). In this study, the immunization with the combined vaccine addressed the defects of inactivated *A. pleuropneumoniae* and RTA protein alone, reducing the clinical symptoms and improving the survival rate of *A. pleuropneumoniae*-infected mice. This approach could improve the immune effect and provide a theoretical basis for developing a comprehensive and safe pleural pneumonia vaccine.

## Conclusion

At present, APP infection is harmful to the pig industry worldwide, and there is a serious problem of cross infection, but there is no effective preventative vaccine. Most *APP* vaccine studies focus on the subunit structure, but research on epitope-based vaccines is still scarce. In this study, we designed an epitope-based vaccine combined with inactivated bacteria to prevent *APP* cross infection. We are the first to describe the design and expression of the RTA protein, which was used to immunize mice in combination with inactivated *APP* and protected them against *APP1* and *APP5b* infection. This study has important reference value and significance for preventing cross infection by *Actinobacillus pleuropneumoniae.*

## Supplementary information

**Additional file 1:** The prediction result of ADH by DNAstar software. (A) The prediction of ADH secondary structure. (B) The antigenicity analysis of ADH.

**Additional file 2:** Expression of the RTA protein under different conditions. (A) Expression of the RTA protein at different temperatures. M, 170 kDa marker; 1, noninduced bacteria; and 2-, 3, and 4, induced at 16, 25, 37 °C, respectively. (B) Expression of the RTA protein at different induction times. M, 170 kDa marker; 1, noninduced bacteria; 2–9, induced for 1–8 h, respectively. (C) Expression of the RTA protein at different IPTG concentrations. M, 170 kDa marker; 1, noninduced bacteria; and 2–8, induced at 0.05, 0.1, 0.5, 1, 1.5, 2, 3 mM IPTG, respectively.

**Additional file 3:** (A), (B), (C) The survival rates of the mice immunized with PBS, RTA protein, inactivated *APP1* alone, inactivated *APP5b* alone, and RTA protein plus inactivated *APP* at 0, 14, and 28 d and challenged with *APP1* and *APP5b* separately at 35 d. The mice were observed for 3 d to record the survival rates.

## Data Availability

All data generated or analysed during this study are included in this published article and its Additional files 1, 2, 3.

## Abbreviations

*A. pleuropneumoniae* /*APP*: *Actinobacillus pleuropneumoniae*
*rta*: Recombinant tandem antigen
RTA: The capital letter of rta
Apx: *Actinobacillus pleuropneumoniae* toxin
TAA: Trimeric autotransporter adhesion
ADH: The head domain of TAA
*P. acnes*: *Propionibacterium acnes*
*E. coli*: *Escherichia coli*
BHI: Brain heart infusion
PBS: Phosphate-buffered saline
BCA: Bicinchoninic acid
SDS-PAGE: SDS-polyacrylamide gel electrophoresis
HRP: Horseradish peroxidase
OMP: Outer membrane protein
