# Supplementary figures and images for: Recombinant tandem epitope vaccination provides cross protection against Actinobacillus pleuropneumoniae challenge in mice
Source: AMB Express. 2020 Jul 8;10:123. doi: 10.1186/s13568-020-01051-1 (PMC7341470; doi:10.1186/s13568-020-01051-1)

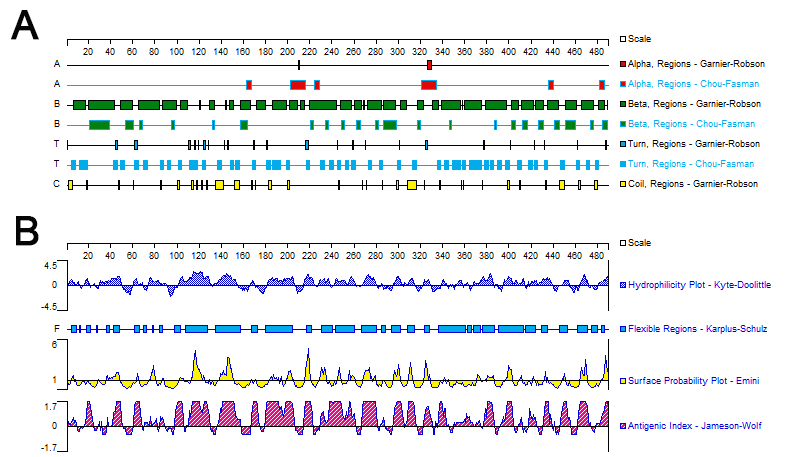

Supplement: Supplementary file 1 — Additional file 1: The prediction result of ADH by DNAstar software. (A) The prediction of ADH secondary structure. (B) The antigenicity analysis of ADH. [file 13568_2020_1051_MOESM1_ESM.tiff]

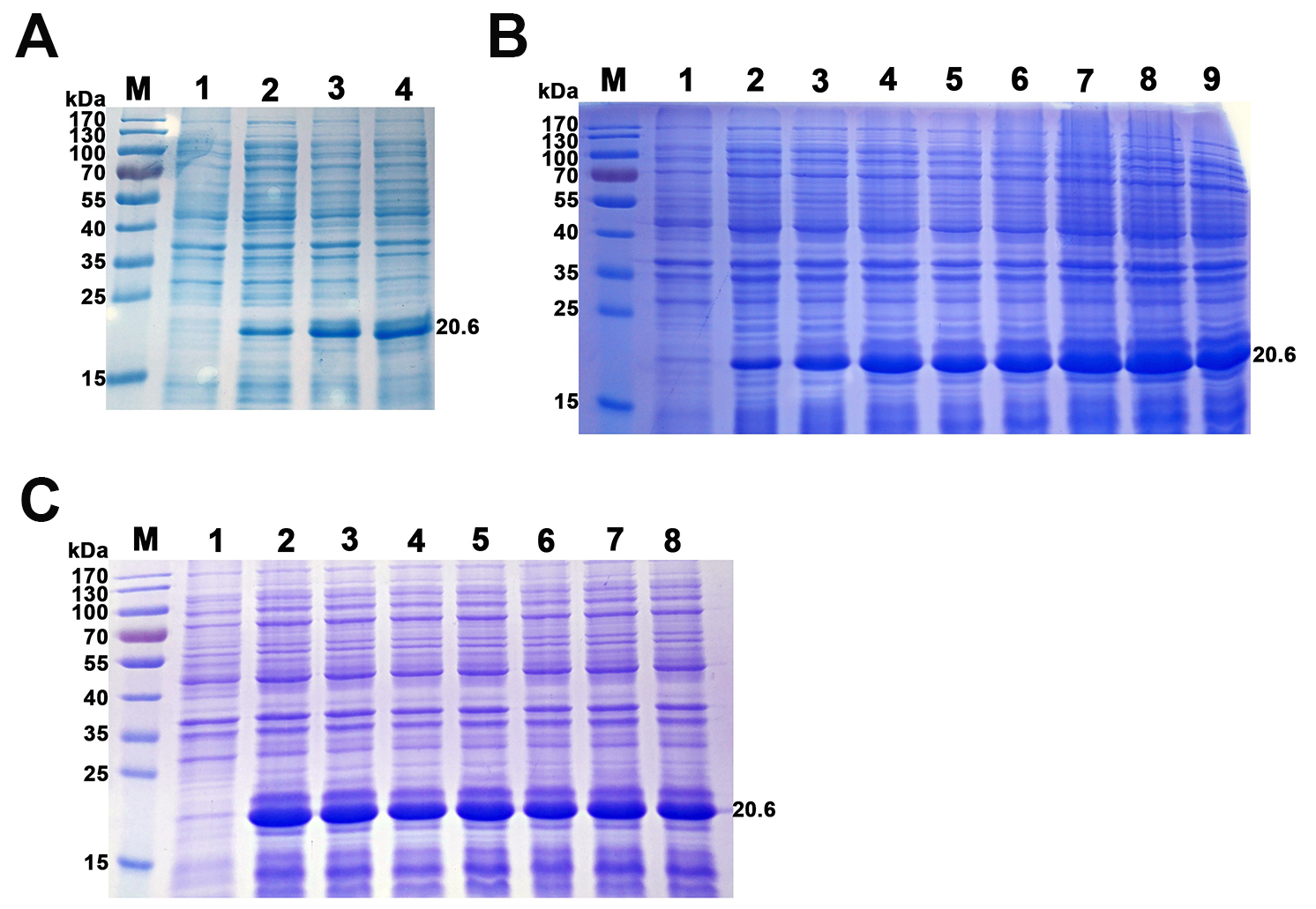

Supplement: Supplementary file 2 — Additional file 2: Expression of the RTA protein under different conditions. (A) Expression of the RTA protein at different temperatures. M, 170 kDa marker; 1, noninduced bacteria; and 2-, 3, and 4, induced at 16, 25, 37 °C, respectively. (B) Expression of the RTA protein at different induction times. M, 170 kDa marker; 1, noninduced bacteria; 2–9, induced for 1–8 h, respectively. (C) Expression of the RTA protein at different IPTG concentrations. M, 170 kDa marker; 1, noninduced bacteria; and 2–8, induced at 0.05, 0.1, 0.5, 1, 1.5, 2, 3 mM IPTG, respectively. [file 13568_2020_1051_MOESM2_ESM.tiff]

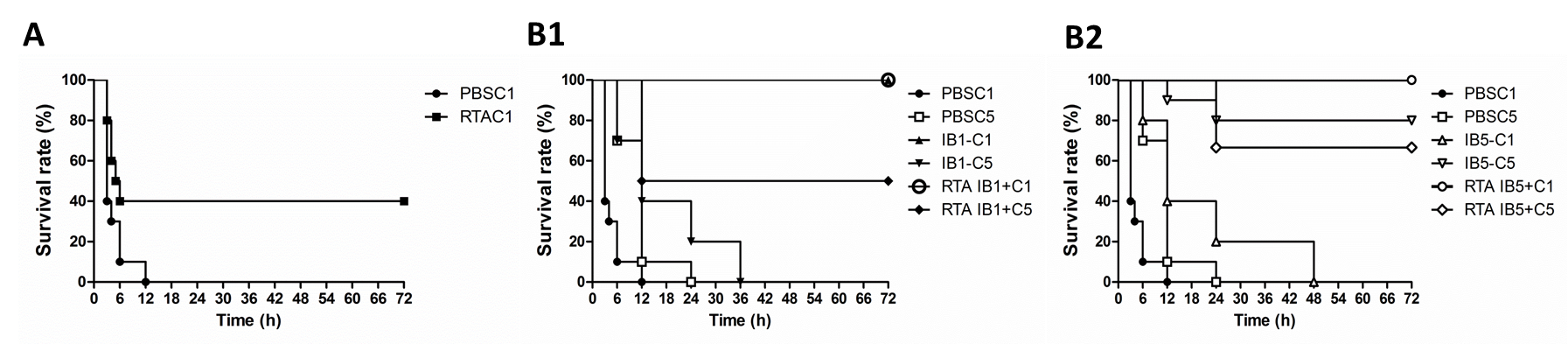

Supplement: Supplementary file 3 — Additional file 3: (A), (B), (C) The survival rates of the mice immunized with PBS, RTA protein, inactivated APP1 alone, inactivated APP5b alone, and RTA protein plus inactivated APP at 0, 14, and 28 d and challenged with APP1 and APP5b separately at 35 d. The mice were observed for 3 d to record the survival rates. [file 13568_2020_1051_MOESM3_ESM.tiff]
